# Role of family factors in provision and perception of social support for older people in Iran: a cross-sectional survey

**DOI:** 10.1186/s12875-023-02236-w

**Published:** 2023-12-19

**Authors:** Maryam Tajvar, Emily Grundy, Astrid Fletcher, Elizabeth Allen, Badriyeh Karami

**Affiliations:** 1 Department of Health Management, Policy and Economics, School of Public Health, Tehran University of Medical Sciences, Tehran, Iran; 2 Institute for Social and Economic Research, University of Essex, Essex, UK; 3 Faculty of Epidemiology & Population Health, London School of Hygiene and Tropical Medicine, London, UK; 4 Behavioral Diseases Research Center, Health Institute, Kermanshah University of Medical Sciences, Kermanshah, Iran

**Keywords:** Family, Social Support, Older people, Iran

## Abstract

**Background:**

Iran has experienced a very fast fertility transition. The process of demographic transition, coupled with modernization, has had considerable consequences for the structure and function of families. There is rising concern in Iran about a potential decline in family care and support for older people as a result of these changes. The main aim of this study was to provide a benchmark by examining current associations between family factors and older people’s social support, both perceived and received.

**Methods:**

A cross-sectional survey of a random sample of 644 people aged 60 + years resident in Tehran was conducted using stratified cluster random sampling method in 2015. Outcome variables were perceived social support, as measured by Social Provision Scale, and received instrumental social support. Multilevel mixed-effects models were used to examine the hypotheses.

**Results:**

The analyses showed that most of the family factors measured, including family size (p = 0.01), living arrangements (p = 0.05), and amount of contact with family members (p = 0.001) were associated with older people’s receipt of instrumental social support. Living arrangements and quality of relationships with family members were associated with older people’s perceptions of social support (p < 0.001). Also, a significant gender interaction was found in associations between family size and SPS (p = 0.03). Having a large size family was positively associated with higher SPS for women (Coef. = 3.9, p = 0.009) but not for men (Coef. = -0.4, p = 0.7).

**Conclusion:**

findings of this study support the premise that most of family factors play an important role in provision and perception of social support for Iranian older people. Further policies should mostly be selective of those at higher risk of low support such as widowed, childless, those living alone, having poor relationship with their relatives and those with worse health status. The results of this study may be utilized to target older populations who are at higher risk of low support with innovative programs that focus on building social networks and enhancing social support.

## Introduction

Iran has experienced one of the fastest fertility transitions in the world. The Total Fertility Rate (TFR) decreased from 7 in 1980 to 2.5 in 1996 and further to 1.6 in 2016 and remaining stable until now [1, 2]. This rapid demographic transition has made Iran one of the fastest-aging countries in the world. It is projected that the share of people aged 65 + in the total population of Iran will increase from just 4% in the year 2000 to over 25%, according to the United Nations (UN) low variant projection, by 2050 [3]. The demographic transition process coupled with very major changes in the governance, economy, cultural, and socio-economic context has had considerable consequences for many aspects of Iranian society [4], including changes in the composition and functions of the family [5]. ‘Family’ is conceptualized by Iranian officials as comprising those individuals who are related through parental (vertical) or marital (vertical or lateral) relationships, including members living in the same household and those living elsewhere [6]. A family, by this definition, which is adopted in this research, includes an individual’s parents, children, spouse, in-laws, siblings, grandparents and grandchildren. There is a rising concern in Iran about a potential decline in the care and support of older people by the family as a result of these demographic and social changes. Due to considerable decline in the number of children, particularly for older people in next decade, it is timely to consider how family characteristics of older people and their children are associated with their social support [7, 8].

Social support is an exchange of resources between at least two individuals which is perceived by the provider or the recipient to be intended to enhance the well- being of the recipient [9]. Social support has two important dimensions: ‘perceived’ and ‘received’ social support. ‘Perceived social support’ refers to one’s perception of potential access to social support, whereas ‘received social support’ refers to the reported receipt of support resources, usually during a specific time period [10]. Social support is of particular importance for older people, as later life is associated with an increased risk of exposure to various stressors such as the onset of chronic conditions and functional limitations, loss of sources of income, and loss of spouse and confidants [11]. As there is evidence that social support is associated with the health of Iranian older people [12, 13], there is a concern that the well-being of older people, may adversely be affected if smaller family sizes lead to a reduction in their ‘perceived’ and ‘received’ social support.

The results of previous research show that the number of children is an important determinant of support [14, 15], but future reductions in support may not be as dramatic as anticipated [14]. In addition, older people in three-generation households had better mental well-being than older adults in single-generation households in China. Receiving financial support from adult children increased well-being and well-being improved with good emotional cohesion with children [16]. The primary objective of this study was to examine associations between family factors of older people and their social support. As there is inconsistency in literature on the effect of family factors on the social support of men and women differently [17–19], we have also been interested to explore gender interactions in any association between family factors and social support; and to examine other socio-demographic factors associated with differentials in different dimensions of social support.

## Methods

### Study design

A cross-sectional survey of a representative sample of community-resident older people aged 60 + years in Tehran was conducted in 2015 to address study questions. Tehran is the largest and capital city and located at the north centre of Iran. Human Development Index (HDI) of Tehran has been calculated as high at 0.810 in 2021 [20]. Tehran is divided into 22 districts and 374 neighbourhoods, which are embedded one in another.

**Fig. 1:**
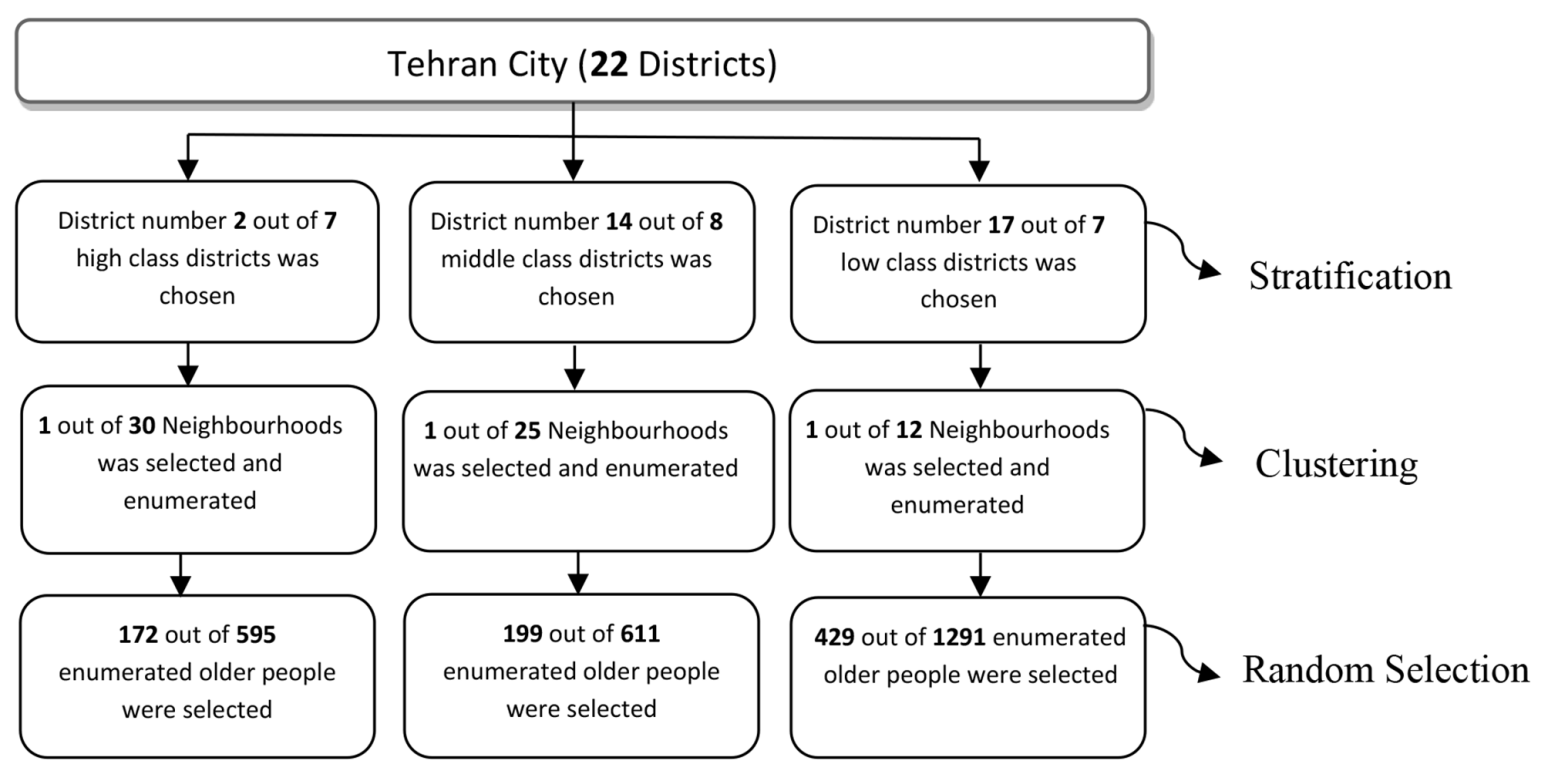
Multistage stratified cluster sampling strategy with the PPS allocation method in this study

The required sample size was calculated using EPI Info software at 800 based on an alpha level of 0.05 and power of 80% using an expected odds ratio (OR) of 2 and a possible non-response of 10% [21] and a design effect of 1.5, based on results from earlier studies [22, 23]. This study used a multistage stratified cluster sampling strategy with probability proportionate to size (PPS) allocation method within study clusters to ensure representation of people from neighbourhoods of different socio-economic status (SES). In order to make the sampling frame for the study, household enumeration was undertaken to identify all those aged 60 and over in the selected neighbourhoods. Older people who refused to participate in the study (102 cases out of 2497) in the enumeration stage were excluded from the frame. The stages of sampling are shown in Fig. 1.

The data were collected using face-to-face structured interviews to complete a structured multi-sectional questionnaire at participants’ homes, taking each about 30 min in average. Three fieldworkers conducted the data collection after training in the field and passing a pilot stage, used for testing the study questionnaire, to identify problems in the process of data collection and to test the performance of fieldworkers.

In total 644 people responded (76% response rate). There was no systematic difference between main characteristics of non-respondents compared with respondents. Ethical approval for the study was given by the ethical committee of the London School of Hygiene and Tropical Medicine (LSHTM) and the ethical committee of the Tehran University of Medical Sciences (TUMS) (Ethics approval code: 8904-27-02-88).

### Measurement of study variables

A structured multi-sectional questionnaire was used to collect study data. In this study, “family factors” were considered as independent variables and “social support” as dependent variable. Further variables were also measured as covariates, required in testing the study hypothesis. In addition to measurement method of these variables provided below, further information also is available in our previous publication [13].

### Social support

In this study both perceived and received social support has been applied. The Farsi version of the Social Provisions Scale (SPS) translated and validated in Iran by Zaki (2009) [24], was used to measure perceived social support. Cronbach’s alpha for this scale for all participants, males and females were reported as 0.85, 0.87 and 0.82 respectively. The SPS measures 6 functions of social support and includes 24 items with a four-point Likert scale ranging from “strongly disagree” to “strongly agree” and higher scores indicating a higher degree of perception of SPS (score ranging 24–96).

To measure received ISS, we included a number of questions derived from a review of the literature, previous scales and testing during a pilot study. ISS has information on types of support received (transportation support, housework support, paperwork support, financial support), and from whom (anyone, spouse, children). We created a summary continuous indicator of ISS, derived using Explanatory Factor Analysis (EFA) in STATA for use in the multivariable analysis. Higher scores on this factor showed a higher probability of receiving ISS.

### Family factors

A large number of questions were included about ‘family factors’ including family structure and characteristics, living arrangements, and quantity and quality of relationships with family members. A grid was created for recording family factors, including for each participant a list of all living family members which included *age, gender, number of children, marital status, economic status, employment*, and *education* of members listed. From this grid, information on family size, household size, the structure and characteristics of family members and also living arrangements was extracted. *Living arrangements*, which refers to those people living together, was recorded using a question located on the family information grid, “where does each family member live?” This question was used to identify co-resident family members and the geographic distance between participants and family members who lived elsewhere. The quality and quantity of relationships between older people and their family network were measured using two questions for each family member: “how often do you meet?” and “how close is the relationship?”. The first question, had 6 answer options ranging from every day to not at all. Because most of the people reported everyday meeting, we dichotomised the answer options in our results as every day and less than every day. The second question also had 5 answer options ranging from very good to bad. Again, as most people responded very good, we dichotomised the responses as very good and less than very good.

### Other covariates

A range of covariates were selected based on literature including age, gender, educational level, economic status, physical health, as measured by the Nagi scale [25] and mental health, as measured by the General Health Questionnaire (GHQ) scale. It was hypothesized that these selected covariates might confound mediate or moderate the associations between family factors and social support, thus they had to be measured and their effects had to be adjusted in the multivariable analyses.

### Data analysis

After collecting the data, the information from the 644 completed questionnaires was coded and entered into STATA Release 14 for analyses. The data collected were clustered and had a hierarchical structure. Thus, multivariable ‘mixed effects modelling’ was selected as the most appropriate. As outcome variables in this study, including the SPS Score and the ISS score, were continuous, *multilevel mixed-effects linear regression models* were used by the *xtmixed* command in STATA. Normal distribution and linearity of the outcome variables were checked before analysis. To check for normality, in addition to eyeballing the histograms, tests of normality (e.g. *sktest, swilk* and *sfrancia* in STATA) were also run. The linearity assumption for linear regression was tested by performing a scatter plot of the independent versus outcome variable. As a result, histograms and statistical tests showed fairly normal distribution of both outcome variables, suggesting that the ISS score, as an outcome variable, could be used in its original continuous form in multivariable analysis.]

For analysing the data in this study and test the study hypothesis, four models were run; in the first model (crude analysis), the association of family factors with social support (dependent variable) were initially assessed one by one in a mixed-effect model adjusted only for age and gender. The selection of variables was based on the conceptual approach informed by theoretical considerations and results of the literature review. In the second model, only selected family variables were included in the model, based on the result of the Model 1; again, adjusted only for age and gender. The third model was the same as Model 2, but other covariates were added to check how these affected the association between main independent and dependent. In all models, only people with complete data on all variables were included. Finally, Model 4 which was similar to the previous model but with an additional section to check the possible gender interaction was performed. All variables were entered to the main models after checking collinearity.

## Results

### Description of characteristics of older people, their family factors and social support status

Characteristics of study members and their families including age, gender, education, economic status, their health status (GHQ and Nagi scores) and a list of family factors are described in Table 1. Men and women comprised exactly the same number in this study (322 men and 322 women), and the youngest age group (60–69) comprised more than half of the participants (51%) and 71% were married. The average household size of participants was 3 people (including the respondent). Just over half the participants (54%) were living in the low-class area, 27% in the middle-class area and 19% in the high-class area. Almost half (48%) the participants were illiterate and illiteracy was twice as high among women. The mean (SD) score for the SPS was 71.8 (9.7) with a range of 24–96. The mean (SD) score of ISS for men and women was similar (men: m = 3.25, SD = 0.66, women: m = 3.24, SD = 0.65) (p = 0.85). More detailed descriptive information on study variables are available in previous publications from same study [12, 13].

**Table 1:** Distribution of participants by demographic, socio-economic and family characteristics, by gender

Variables	All (n = 644)	Men (n = 322)	Women (n = 322)
Age			
Mean (SD)	69.8 (7.2)	70.6(7.5)	68.9(6.8)
Education			
Illiterate	307(47.7)	104(32.2)	203(63.0)
Preliminary (1–5)	215(33.4)	130(40.3)	85(26.3)
Second level	35(5.4)	26(8.0)	9(2.7)
Diploma	41(6.3)	25(7.7)	16(4.9)
University qualification	41(6.3)	33(10.2)	8(2.4)
Religion degree	4(0.6)	4(1.2)	0(0.0)
Economic status perceived			
Dependent on others (no income)	105(16.3)	41(12.2)	64(19.8)
Enough for living	534(82.9)	276(85.7)	258(80.1)
More than enough	5(0.7)	5(1.5)	0(0.0)
Family size			
0–9 members	76 (11.8%)	-	-
10 + members	568 (88.2%)	-	-
Household size			
1 (only participant)	79 (12.1%)	-	-
2–4 people	449 (69.7%)	-	-
5 people or more	116(18.0%)	-	-
Mean (SD)	3.0(1.7)	3.3(1.7)	2.7(1.6)
Marital status			
Married	459(71.2)	290(90.0)	169(52.4)
Widowed	177(27.4)	30(9.3)	147(45.6)
Other (never married, divorced)	8(1.2)	2(0.6)	6(1.8)
N of children (n = 2993)			
Mean (SD)	4.6 (2.1)	4.7 (2.0)	4.6 (2.2)
N of daughter			
Mean (SD)	2.2(1.4)	-	-
N of son			
Mean (SD)	2.3(1.3)	-	-
Having 1 + rich child (n = 2993)			
No (n %)	2658 (88.8)	-	-
Yes (n %)	335(11.3)	-	-
Having 1 + highly educated child (n = 2993)			
No	2273 (75.9)	-	-
Yes	720(24.4)	-	-
Living arrangement			
Living alone/others	94(14.5)	14(4.3)	80(24.8)
Living with children only	101(15.6)	24(7.4)	77(23.9)
Living with spouse only	174(27)	99(30.7)	75(23.2)
Living with spouse& children	275(42.7)	185(57.4)	90(27.9)
Geographic proximity of 1 + child ***			
Same home	376(58.3)	209(65.9)	167(53.8)
Same neighbourhood	116(17.4)	50(15.7)	66(21.2)
Same city or further	152(23.6)	63(18.4)	89(25.0)
Quantity of contacts with 1 + child***			
Less than everyday	181(28.2)	81(24.0)	100(28.4)
Everyday	463(71.8)	241(76.0)	222(71.6)
Quality of relationships with spouse***			
Less than very good	306(47.6)	97(22.5)	209(35.2)
Very good	338(52.4)	225(77.5)	113(66.8)
Quality of relationships with 1 + child***			
Less than very good	193(30.0)	93(27.4)	100(28.4)
Very good	451(70.0)	229(72.2)	222(71.6)
Quality of relationships with 1 + family member			
Less than very good	115(17.9)	51(15.9)	64(19.2)
Very good	529(82.1)	271(84.1)	258(80.8)
GHQ score			
Rest quartiles	489(75.9)	273 (84.5)	216 (67.0)
Worst quartile	155(24.1)	49 (15.2)	106 (32.9)
Nagi score			
Rest quartiles	455(70.6)	260 (81.2)	195 (61.5)
Worst quartile	182(29.4)	60 (18.7)	122 (38.5)

### Associations between family factors and perceived social support

Results from the analysis of associations between family-related factors and perceived social support, as measured by the *SPS*, are presented in Table 2. As shown in the Table, after performing initial analysis in Model 1and 2, then in Model 3, the effect of family factors on SPS score, controlling for the effects of individual-level covariates, were tested. The result showed that the association between *living arrangements* and *quality of relationships with family* and *SPS* are highly significant (p ≤ 0.003). Among covariates added in this model, *being female* and having a *higher education* showed a significant positive association with SPS score, whereas *poorer GHQ* showed a significant negative association with SPS. *Age, economic status* and *Nagi score* were not significantly associated with SPS in this model. In a separate model (results not shown) I also repeated Model 3 replacing *number of children* with *family size* (since these two variables were highly correlated) to test whether having more children (when fully adjusted for other covariates) was associated with receiving more ISS. However, I found no significant association between having more children and a higher SPS score (Coef. = 0.03, p = 0.01). Moreover, in two separate fully adjusted models (similar to Model 3) I compared whether a high-quality relationship with a spouse appeared more important to SPS than a high-quality relationship with at least one child. The results showed that having a very good relationship with a spouse versus otherwise was not significantly associated with SPS (Coef. =1.7, CI=-0.13, 3.5) whereas there was a significant association between having a very good relationship with at least one child and higher the SPS score (Coef. =2.6, CI = 1.1, 4.2, p = 0.001) (results not shown).

**Table 2:** Mixed-effects linear regression models for analysing the association of family factors and perceived social support (higher scores indicating higher support)

Variables	Model 1**		Model 2		Model3		Model 4	
	Each single Fac.+Age + Gender		Multivariable Family Fac.+Age + Gender		Model 2 + Other Covariates		Model 3 + Interaction of Gender	
	Coef. (95% CI)	P*	Coef. (95% CI)	P	Coef. (95% CI)	P	Coef. (95% CI)	P
**Constant**			76.3 (66.74,86.00)	< 0.001	69.60(59.77,79.43)	< 0.001	72.49(63.47,81,51)	< 0.001
**Family factors**								
**Age**								
Continuous	-0.26(-0.36, -0.16)	< 0.001	-0.18(-0.28, -0.07)	0.001	-0.10(-0.21,0.00)	0.06	-0.09(-0.20,0.01)	0.08
**Gender**								
Man	Ref. 1		Ref. 1		Ref. 1			
Woman	-1.41(-2.8, -0.02)	0.05	0.24(-1.27,1.75)	0.76	1.77(0.17,3.37)	0.03		
**Family size**								
0–10 members	Ref. 1		Ref. 1		Ref. 1			
11 + members	2.31(0.20,4.42)	0.03	0.95(-1.10,3.02)	0.36	1.51(-0.52,3.54)	0.14		
**Household size**								
1 (only participant)	Ref. 1							
2–4 people	4.40(2.03,6.78)	0.001						
5 people or more	3.85(1.00,6.71)							
**Married or not**								
No	Ref. 1							
Yes	5.42(3.64,7.20)	< 0.001						
**N of children**								
Continuous	0.22(-0.13,0.58)	0.22						
**N of daughter**								
Continuous	0.02(-0.47,0.51)	0.92						
**N of son**								
Continuous	0.58(0.03,1.13)	0.04						
**Having 1 + rich child**								
No	Ref. 1							
Yes	0.44(-1.41,2.29)	0.64						
**Having 1 + highly educated child**								
No	Ref. 1		Ref. 1		Ref. 1		Ref. 1	
Yes	2.36(0.78,3.94)	0.003	1.36(-1.18,2.90)	0.08	0.49(-1.05,2.04)	0.53	0.45(-1.09,1.99)	0.57
**Living arrangement**								
Living alone/others	Ref. 1		Ref. 1		Ref. 1		Ref. 1	
Living with child/ren only	1.18(-1.42,3.79)	< 0.001	0.49(-2.08,3.07)	0.0001	0.04(-2.05,2.60)	0.003	-0.11(-2.66,2.44)	0.003
Living with spouse only	5.15(2.72,7.58)		4.07(1.62,6.51)		2.94(0.51,5.37)		2.77(0.35,5.20)	
Living with spouse& child/ren	6.10(3.68,8.52)		4.75(2.31,7.18)		3.57(1.15,5.98)		3.44(1.03,5.85)	
Geographic proximity of 1 + child ***								
Same home	Ref. 1							
Same neighbourhood	-1.32(-3.33,0.67)	0.23						
Same city or farther	0.69(-1.25,2.64)							
**Living with 1 + daughter**								
No	Ref. 1							
Yes	0.46(-1.17,2.10)	0.60						
**Living with 1 + son**								
No	Ref. 1							
Yes	0.69(-0.86,2.24)	0.38						
Quantity of contacts with 1 + child***								
Less than everyday	Ref. 1							
Everyday	1.66(-0.05,3.37)	0.06						
Quality of relationships with spouse ***								
Less than very good	Ref. 1							
Very good	1.70(-0.20,3.61)	0.08						
Quality of relationships with 1 + child***								
Less than very good	Ref. 1							
Very good	3.16(1.57,4.76)	< 0.001						
**Quality of relationships with 1 + family member**								
Less than very good	Ref. 1		Ref. 1		Ref. 1		Ref. 1	
Very good	5.23(3.34,7.11)	< 0.001	4.02(2.12,5.93)	< 0.001	3.48(1.61,5.34)	< 0.001	3.42(1.03,5.85)	< 0.001
**Other covariates**								
**Education**								
Illiterate	Ref. 1				Ref. 1		Ref. 1	
1–9 years	2.10(0.40,3.80)	0.0004			1.06(-0.62,2.75)	0.02	1.10(-0.58,2.78)	0.02
10 years and more	5.81(2.90,8.72)				4.11(1.23,7.00)		4.07(1.20,6.94)	
**Economic status perceived**								
Poorer than average	Ref. 1				Ref. 1		Ref. 1	
Same or better than average	2.76(1.15,4.37)	0.001			1.18(-0.39,2.76)	0.14	1.21(-0.36,2.78)	0.13
**GHQ**								
Rest quartiles	Ref. 1				Ref. 1		Ref. 1	
Worst quartile	-6.10(-7.79, -4.40)	< 0.001			-4.54(-6.31,-0.2.7)	< 0.001	-4.58(-6.33, -2.82	< 0.001
**Nagi**								
Rest quartiles	Ref. 1				Ref. 1		Ref. 1	
Worst quartile	-2.76(-4.51, -1.01)	0.002			-1.16(-2.89,0.56)	0.19	-1.13(-2.85,0.59	0.09
Interaction Family size*Gender P.V								0.03
**Family size in men**								
0–10 members							Ref. 1	
11 + members							-0.41(-3.06,2.24)	0.76
**Family size in women**								
0–10 members							Ref. 1	
11 + members							3.89(0.95,6.82)	0.009
LR test vs. linear regression P.v Neighbourhood-level ICC Household-level ICC			< 0.001 0.26 0.72		< 0.001 0.22 0.68		0.0001 0.22 0.68	

* *P*-value of coefficient reported for each dummy variable compared to the baseline category controlled for other variables. For categorical variables overall *P*-value was reported using ‘*testparm’* in STATA. In all models only people with complete data on all variables were included

**In Model 1, ICC for levels of analysis were not reported as separate univariable models were fitted for each variable and each model had different ICC for each level

***For these variables, analysis excluded those without a spouse/ a child (Not applicable)

Additionally, as one of the exploratory objectives was to explore whether associations between family related variables and SPS differed by gender, at first, we ran Model 3 separately for men and women and then ran Model 4 including an interaction term for gender for differences. Results showed that the associations between all variables in Model 3 and *SPS* were very similar for men and women with the exception of the *family size* variable. Accordingly, an interaction term of *gender* and *family size* was added to Model 4 in order to formally test whether the effects of *family size* on *SPS* were modified by the effect of gender in the full model. Results confirmed the hypothesis that the gender interaction was significant (p = 0.03). Having a large size family (11 + members) was positively associated with higher SPS for women (Coef. = 3.9, p = 0.009) but not for men (Coef. = -0.4, p = 0.7). As *family size* was highly correlated with *number of children*, this result also may mean that having more children was important factor in SPS for women but not for men. Having more children also increased the chance of having very good relationships with at least one child, which was already shown to be important in the SPS score. The coefficient of gender was not reported in the interaction model as the result provided by STATA was the effect of gender in one group only.

### Associations between family factors and received instrumental social support (ISS)

As shown in Table 3, after doing preliminary analysis in Model 1 and 2, then in the main model, Model 3, the associations between family factors and ISS score, controlling for the effects of individual-level covariates including *perceived economic status, GHQ* and *Nagi* score were checked. In this model, the association between *family size* and *quantity of contacts with at least one child* with ISS score did not change compared to the previous model, though the association with *living arrangements* was slightly attenuated (overall p = 0.02). In this model, the *economic status* of the individual was not significantly associated with ISS. However, both *GHQ* and *Nagi* score showed a significant association with ISS. Interestingly, poorer *GHQ* had a negative association with ISS (Coef. = -0.16, p = 0.007) but poorer *Nagi* score had a positive association (Coef. = 0.18, p = 0.002) with ISS.

**Table 3:** Mixed-effects linear regression models for analysing the association of family factors and received instrumental social support (higher scores indicating higher support)

Variables	Model 1**		Model 2		Model3		Model 4	
	Each single Fac.+Age + Gender		Multivariable Family Factors + Age+ Gender		Model 2 + Other Covariates		Model 3 + Interaction of Gender	
	Coef. (95%CI)	P*	Coef. (95% CI)	P	Coef. (95% CI)	P	Coef. (95% CI)	P
**Constant**			3.08(2.45,3.71)	< 0.001	3.18(2.51–3.85)	< 0.001	3.16(2.55,3.78)	< 0.001
**Age**								
Continuous	-0.006(-0.01, -0.01)	0.05	<-0.01(-0.01,0.002)	0.22	<-0.01(-0.01,0.00)	0.11	<-0.01(-0.01,0.00)	0.11
**Gender**								
Man	Ref.		Ref. 1		Ref. 1			
Woman	-0.03(-0.12,0.06)	0.49	<-0.01(-0.10,0.09)	0.95	<-0.01(-0.11,0.09)	0.85		
**Family size**								
0–10 members	Ref.		Ref. 1		Ref. 1		Ref. 1	
11 + members	0.22(0.08,0.36)	0.002	0.17(0.03,0.32)	0.01	0.18(0.04,0.32)	0.01	0.18(0.04,0.32)	0.01
**Household size**								
1 (only participant)	Ref.							
2–4 people	0.30(0.14,0.47)	0.0008						
5 people or more	0.32(0.12,0.52)							
**Married or not**								
No	Ref.							
Yes	0.17(0.04,0.29)	0.008						
**N of children**								
Continuous	0.04(0.01,0.06)	0.001						
**N of daughter**								
Continuous	0.02(-0.01,0.05)	0.26						
**N of son**								
Continuous	0.07(0.04,0.11)	< 0.001						
**Having 1 + rich child**								
No	Ref.							
Yes	0.05(-0.07,0.17)	0.44						
**Having 1 + highly educated child**								
No	Ref.							
Yes	0.09(-0.01,0.20)	0.09						
**Living arrangement**								
Living alone/others	Ref.		Ref. 1		Ref. 1		Ref. 1	
Living with children only	0.31(0.13,0.49)	0.0006	0.16(-0.03,0.35)	0.01	0.12(-0.07,0.31)	0.02	0.11(-0.08,0.31)	0.05
Living with spouse only	0.30(0.14,0.47)		0.27(0.10,0.43)		0.24(0.07,0.41)		0.23(0.06,0.40)	
Living with spouse& children	0.33(0.16,0.49)		0.16(-0.02,0.34)		0.13(-0.05,0.31)		0.12(-0.06,0.31)	
Geographic proximity of 1 + child ***								
Same home	Ref.							
Same neighbourhood	0.02(-0.12,0.15)	0.15						
Same city or farther	-0.12(-0.25,0.01)							
**Living with 1 + daughter**								
No	Ref.							
Yes	0.08(-0.03,0.20)	0.15						
**Living with 1 + son**								
No	Ref.							
Yes	0.08(-0.03,0.18)	0.15						
Quantity of contacts with 1 + child***								
Less than everyday	Ref.		Ref.		Ref.		Ref.	
Everyday	0.19(0.07,0.30)	0.001	0.23(0.09,0.37)	0.001	0.22(0.08,0.36)	0.002	0.21(0.07,0.35)	0.003
Quality of relationships with spouse ***								
Less than very good	Ref.							
Very good	-0.08(-0.20,0.04)	0.21						
Quality of relationships with 1 + child***								
Less than very good	Ref.							
Very good	-0.06(-0.17,0.04)	0.25						
**Quality of relationships with 1 + family member**								
Less than very good	Ref.							
Very good	-0.04(-0.17,0.09)	0.53						
**Education**								
Illiterate	Ref.							
1–9 years	0.06(-0.05,0.17)	0.22						
10 years and more	-0.07(-0.26,0.12)							
**Economic status perceived**								
Poorer than average	Ref.				Ref. 1		Ref. 1	
Same or better than average	0.12(0.00,0.22)	0.03			0.08(-0.02,0.19)	0.13	0.08(-0.02,0.19)	0.11
**GHQ**								
Rest quartiles	Ref.				Ref. 1			
Worst quartile	-0.18(-0.30,-0.06)	0.002			-0.16(-0.28,-0.04)	0.007		
**Nagi**								
Rest quartiles	Ref.				Ref. 1		Ref. 1	
Worst quartile	0.13(0.01,0.25)	0.02			0.18(0.06,0.30)	0.002	0.18(0.06,0.30)	0.003
Interaction GHQ*Gender P.V								0.076
**GHQ in men**								
Rest quartiles							Ref. 1	
Worst quartile							-0.04(-0.22,0.14)	0.67
**GHQ in women**								
Rest quartile							Ref. 1	
Worst quartile							-0.23(-0.38,-0.09)	0.001
LR test vs. linear regression P.v Neighbourhood-level ICC Household-level ICC			< 0.001 0.03 0.16		< 0.001 0.03 0.16		< 0.001 0.03 0.16	

* *P*-value of coefficient reported for each dummy variable compared to the baseline category controlled for other variables. For categorical variables overall *P*-value was reported using ‘*testparm’* in STATA. In all models only people with complete data on all variables were included

**In Model 1, ICC for levels of analysis were not reported as separate univariable models were fitted for each variable and each model had different ICC for each level

***For these variables, analysis excluded those without a spouse/ a child (Not applicable)

The Model 3 was also repeated substituting *number of children* for *family size* (since these two variables were highly correlated) to test whether having more children (when fully adjusted for other covariates) was associated with receiving more ISS as *family size* did. The results showed a significant association between having more children and receiving more ISS (Coef. = 0.03, p = 0.01) (results not shown). Furthermore, given a rather high correlation between *frequency of contact with children* and *geographic proximity* (r=-0.66), it was interesting to check whether the *geographic proximity of children* had a similar association with ISS score as the *frequency of contacts* with them had. The results showed that despite a rather high correlation, *geographic proximity* of children did not show any significant association with ISS (p = 0.1) when this was substituted for *quantity of contact* with children in the final model. Thus, the important factor regarding children in relation to ISS was ‘contact’ not ‘residence’ (results not shown).

Additionally, gender-specific analysis prior to Model 4 showed that men and women differed considerably in the association of their *GHQ* with ISS. The interaction of gender with *GHQ* was tested formally in Model 4 and results of the interaction analysis showed that women with poor *GHQ* reported significantly poorer ISS compared to other women, while a similar association was not observed for men. However, the interaction of gender with *GHQ* in association with ISS was not significant (p = 0.07).

## Discussion

In accordance with the hypothesis, availability of a spouse, irrespective of gender, showed a very strong positive association with SPS and a borderline association with ISS. These findings are consistent with previous research in Iran by Rambod and Rafiee (2008) [7] who reported that married older people had higher perceived social support than the non-married. Other finding reported earlier indicated the relative importance of the spouse in comparison with children in perception of social support. In particular, for women being widowed may imply the loss of a separate identity. Although widowhood is more common for women than men, the loss of spouse may however have more negative effect on social support of older men as it was shown that ‘wife’ was reported to be the main provider of support for ‘husband’ and thus marriage appears to be more beneficial for older men [26].

Also, findings of this study suggest that the availability and number of children, irrespective of their characteristics, is an important factor associated with the provision of support for older people in contemporary Iran. Moreover, our study did not provide evidence for a significant influence of an extensive family network in increasing SPS, irrespective of the gender of older people, but considering their gender, it was found that having a larger family size (more children) was very important for women’s SPS but not for men’s. Women relied on their children for most types of ISS, thus, availability of children is important for their perceived support too. The less important role of children in SPS of men may reflect the fact that men’s higher power in the family, particularly in the current cohort of older people, leads them, even in old age, to underestimate children’s role and significance in their life, and to keep their dominant role in the family and living independently from their children.

Larger families and more children imply larger networks and potentially offer more social interaction and a higher chance of getting help for older people. Research suggests that co-residence with children as well as the likelihood and amount of support from non-co-resident children depends in part on the number of children available to provide such support [27]. However, it is also possible that a larger family or household creates more conflict, due to the pressures and responsibilities stemming from a larger number of relationships [28], so it cannot be assumed that more children necessarily mean more support. A previous qualitative study of Indonesia found that family size did not seem to be a particularly important influence of the care and support received by older people [29]. Also, there is some evidence that childless older people may be able to draw on other sources of support, such as from other relatives and friends which, if this was sufficient to ‘compensate’ for the absence of children, would also mean that there might be no association between number of children and support [30, 31].

Looking at the results by gender of children showed that having more sons is associated with higher received ISS, but having more daughters is associated with lower received ISS. In Asia gender differences in the provision of support for parents is variable. For example, in India [32] and Korea [33] sons are the main providers of support for their mother; while, in Thailand, daughters (usually the youngest) are more preferred to receive support from elders [34]. In western countries, typically daughters provide emotional support while sons provide financial support [35].

With regard to the living arrangements, it was found to be important for both SPS and ISS. Participants living with both spouse and children had the highest SPS score. However, co-residing only with children had limited role in support of older people. These findings may imply that the supportiveness and helpfulness of children rather than their co-residence is important for support of parents. On the other hand, based on the evidence, living alone is associated with feelings of loneliness, and there is a greater chance that in case of a crisis or accident urgent needs for assistance remains unnoticed [36].

In this research, apart from living arrangements, we also investigated the effects of geographic proximity of children and the amount of contact with them with perception and provision of support of older people. Our analysis revealed that proximity of residence of children was not important for neither the support dimensions, whereas frequency of contact with them was a significant factor for provision of support of older people. These findings suggest that children living in close distance did not necessarily make more visits to parent and that provision of support is associated with ‘visit’ not ‘residence’. Thus, it is possible that non co-residence children even provide more support and care for their parents in Iran. Also, according to the analysis, a higher proportion of women than men had daily contact with children possibly reflecting greater need, for example because of poorer health status; a higher proportion of living alone; widowhood; having more free time (15% of men versus less than 1% of women were working); and closer emotional bonds in line with well-established gender differences in social interaction.

Descriptive results showed that older people reported generally a very good relationship with most of their family member. The study of Zamanzadeh [37] in Iran also showed that among all aspects of social support, emotional support was the highest type provided for older people. Further analysis indicated that having a very good relationship with at least one child made a significant difference to perception of support, but there was no such evidence for the quality of relationship with spouse. Taking into account the evidence of the importance of the availability of a spouse for perception of support in contrast to the availability of more children may suggest the intrinsic value of spouse for higher perception of support, irrespective of quality of relationship with him/her, while the value of children for perception of support depended on their supportiveness and having good relationship with them.

Furthermore, the results showed that poorer *GHQ* had a negative association with ISS but poorer *Nagi* score had a positive association with ISS. This may suggest reverse causality between *GHQ* and ISS, so that less provision of ISS might lead to poor *GHQ* rather than other way around. It is also possible that participants with more depressive symptoms perceived the amount of ISS they received differently and under reported the amount of help they received. However, it is to be expected that when older people have poor functioning (low Nagi score), they need more ISS and so the participants’ ISS increased with more physical limitations.

The main limitation of this study is its cross-sectional design, as in this study design, the temporal associations between family factors and ISS and SPS cannot be ascertained and reverse association cannot be excluded in some cases. Additionally, the results of this study are generalizable only to community- living older people, but not to institutionalized older people, and those hospitalized at the time of the survey or older people living in other parts of Iran. Future studies should include the excluded groups for whom associations may differ and have a wider geographical scope.

Despite the methodological limitations discussed above, the study is of significance. The findings of this study make an important contribution to the scarce research evidence on family support and health of older people in Iran. This research is based on a randomly selected population-based sample and relatively large survey with a high response rate comprehensively measuring a wide range of family and social support measures in one study, together with contextual variables. Moreover, multi-level modelling techniques were used to account for the structure of the data and control for a wide range of confounders. These methods provided a stringent statistical assessment of the association between family and social support.

## Conclusion

This study was conducted to investigate how family structure and characteristics are associated with the provision and perception of support of older people. The research aimed to provide evidence for Iranian authorities and policymakers as well as advancing the study of a neglected topic in Iran. Due to the current demographic and social changes in Iran and its important consequences for future older people, a greater understanding of current associations between family factors and the support and well-being of older people is important to provide invaluable insights into the implications of these changes in the future. This information was fundamental to informed planning for the future.

In summary, findings of this study support the premise that most of the family factors, including family size, living arrangements, and amount of contact with family members are associated with received ISS and living arrangements and a high-quality relationship with family members are associated with perceived support of older people. Based on the findings, it seems that the policies for current older people should mostly be selective of those at higher risk of low support such as widowed, childless, those living alone, having poor relationship with their relatives and those with worse health status. The results of this study also may be utilized to target older populations who are at higher risk of low support with innovative programs that focus on building social networks and enhancing social support.

## Data Availability

the data are available from corresponding author in reasonable request.

## Abbreviations

SPS: Social Provisions Scale
ISS: Instrumental Social Support
LSHTM: The London School of Hygiene and Tropical Medicine
TUMS: Tehran University of Medical Sciences
EFA: Explanatory Factor Analysis
GHQ: General Health Questionnaire
Coef: Coefficient
